# Solitary vulvar metastasis from early-stage endometrial cancer: Case report and literature review

**DOI:** 10.1097/MD.0000000000025863

**Published:** 2021-06-04

**Authors:** Vincenzo Dario Mandato, Valentina Mastrofilippo, Andrea Palicelli, Monica Silvotti, Silvia Serra, Lucia Giaccherini, Lorenzo Aguzzoli

**Affiliations:** a Unit of Obstetrics and Gynecology; b Unit of Surgical Gynecol Oncology; c Unit of Pathology; d Unit of Radiology; e Unit of Radiation Oncology, Azienda USL-IRCCS, Reggio Emilia, Italy.

**Keywords:** endometrial cancers, laparoscopy, recurrence, survival, vulvar metastasis

## Abstract

**Rationale:**

Endometrial cancer (EC) is the most common gynecological malignancy in developed countries. It is usually diagnosed at early-stage and presents a favorable prognosis. Conversely, advanced or recurrent disease shows poor outcome. Most recurrences occur within 2 years postoperatively, typically in pelvic and para-aortic lymph nodes, vagina, peritoneum, and lungs. Vulvar metastasis (VM) is indeed anecdotal probably because of the different regional lymphatic drainage from corpus uteri.

**Patient concerns:**

A 3 cm, reddish, bleeding lesion of the posterior commissura/right labia was found in a 74-year-old woman treated with radical hysterectomy, surgical staging, and adjuvant radiotherapy 1 year before for a grade 2 endometrioid type, International Federation of Gynecology and Obstetrics Stage IB. Vulvar biopsy confirmed the EC recurrence. Pelvic magnetic resonance imaging and positron emission tomography excluded other metastases so VM was radically resected.

**Diagnosis:**

Postoperative histopathology confirmed the diagnosis of grade 2 EC VM.

**Interventions:**

A radical excision of VM was performed.

**Outcomes:**

Patient died from a severe sepsis 27 months after first surgery.

**Lessons:**

Vulvar metastases can show different appearance, occurring as single or diffuse lesions on healthy or injured skin. The surgical approach seems not to influence the metastatic risk, but tumor seeding and vaginal injuries should be avoided. Whether isolated or associated with recurrence in other locations, vulvar metastases imply poor prognosis despite radical treatment. Therefore, any suspected vulvar lesion arisen during EC follow-up should be biopsied and monitored closely, despite that the vulva represents an unusual metastatic site.

## 1 Introduction

Endometrial cancer (EC) is the most common gynecologic malignancy in developed countries.^[1]^ When diagnosed at early stage, it usually has favorable prognosis (77% 5 year overall survival [OS]); conversely, advanced or recurrent disease results in low response to chemotherapy and poor outcome.^[2]^ Sixty-four percent of recurrences usually occur within 2 years postoperatively (87% < 3 years), typically involving pelvic and para-aortic lymph nodes, vagina, peritoneum, or lungs; unusual localizations include abdominal organs/wall, bones, brain, and skeletal muscle.^[3]^

Vulva seems the site of the female genital tract least affected by secondary gynecologic and nongynecologic tumors: vulvar metastases (VMs) represent 5% to 8% of all vulvar cancers and the tumor origin remains unknown in about 10% of the cases.^[4,5]^ VMs from EC are anecdotal, probably because of the different regional lymphatic drainage of uterus (to pelvic and paraaortic lymph nodes) and vulva (to superficial inguinal lymph nodes); thus, EC cells in inguinal lymph nodes are considered distant metastasis. We report a vulvar EC recurrence in a patient treated with total laparoscopic hysterectomy, comprehensive surgical staging, and adjuvant radiotherapy 1 year before. We also performed a literature review discussing the different mechanisms of VM pathogenesis (Table 1).^[5–14]^

**Table 1:** Clinical features of 19 patients with vulvar metastasis from endometrial cancer: review of the English literature including our case.

Author Year	Age	Type of EC	GRA	ST	Neo adju. Ther.	Surgery	Adju Ther.	First recurr. (mo.)	Vulvar site	Other site of recurr.	Size of recurr. (cm)	Symptoms of vulvar recurr.	Surgical treatment of recurr.	Chem.	Radioth.	Horm. Ther.	Time from first to second recurr. (mo)	Site of second recurr.	Overall Survival from diagnosis (mo)	Survival from I° recur (mo)	Status
Dehner LP^[5]^; 1973	56				^∗^ERT,BRT	NO	NO	12	Labium major		1	Painful nodule	NO	NO	YES	NO	14		26	14	DOD
	78			IV	^∗^ERT,BRT	NO	IRT	SYNCH	Labium major		6,5	Vaginal bleeding	NO	NO	NO	NO	5		5	5	DOD
	69				^∗^ERT,BRT	NO	NO	4	Labium major and Clitoris			Painful nodule	NO	NO	YES	NO	10		14	10	DOD
	71				ERT,BRT	TAH, BSO	NO	5	Labium major		1	Painful nodule	NO	NO	YES	NO	5		10	5	DOD
Mazur MT et al^[5]^;1984	NR	NR	NR	NR	NR	NR	NR	NR	NR	NR	NR	NR	NR	NR	NR	NR	NR			NR	NR
Neto AG et al^[6]^; 2003	62								Labius minus			Mass	Wide local excision	NO	YES	NO	0			5	AWD
	63								Labius major			Mass	NO	NO	YES	NO	0			84	NED
	54								Labia majora			Mass	NO	NO	YES	NO	0			9	DOD
	68											Ulceration	NO	YES	NO	NO	0			9	DOD
	51								Labius minus			Ulceration	NO	YES	NO	NO	0			3	DOD
	73								Introitus and Clitoris			Mass	NO	YES	YES	NO	0			41	DOD
Giordano G. et al^[8]^; 2005	66	EH	G3	IIIC		TAH, BSO, PLS	NO	7	Posterior commissura	Liver		Mass	Tumor excision	NO	NO	PG	0		14	7	DOD
Ray K. et al^[9]^; 2006	53	EH	G1	IB		TAH, BSO, SPL, SLL	NO	10	Introitus on marsupialization site		1	Postcoital spotting	Tumor excision	NO	NO	NO	0		26	16	NED
Temkin SM et al^[10]^; 2007	63	SH		IB		TAH, BSO, PLS, LLS	BRT	84	Labium major(exophytic lesion)	Homolateral groin mass	8	Vulvar lesion and groin mass	Radical hemivulvectomy and left groin dissection	YES	YES	NO	12		104	20	DOD
	83	EH	G1	IB		TAH	ERT/BRT on vaginal cuff relapse 5 mlater	36	Right labium major(exophytic lesion)		4	Vulvar itching and bleeding	Radical wide excision with radical groin dissection	NO	NO	NO	0		54	18	NED
Wimmer JL et al^[11]^; 2013	79	EH	G2	IA		TAH, BSO	ERT	5	Right labium major(exophytic lesion)		1	Mass	Partial vulva resection	NO	NO	NO	0		17	12	NED
Fakor F et al^[12]^; 2013	52		G3	IB		TAH, BSO, PLS, LLS	BRT	18	Clitoris		4	Clitoromegally	Wide local excision of clitoris	NO	YES	NO	10		36	18	DOD
Abdullah A et al^[13]^; 2014	87	EH	G1	IB		TLH, BSO, PLS, LLS,	BRT	8	Exophitic lesion at the posterior fourchette		1,9	Physician examination	Partial vulvectomy	NO	NO	NO	0		28	20	NED
Rottenstreich M et al^[14]^; 2019	60	EH	G2	IIIA		TAH, BSO PLS, LLS	CHE	36	Vulva and extending to the pubisand lower abdomen and vaginal vault	Lower abdomen		Painfull lesion	NO	YES	YES	NO	0				NED
Mandato VD et al; 2021	73	EH	G2	IB		TLH, SOB, SPL	ERT/BRT	12	Exophitic lesion of posterior fourchette		1.2×0.9×0.5	Bleeding lesion	Radical resection	NO	NO	NO	10	Liver	27	15	NCD

## 2 Case report

A 73-year-old woman presented with a grade (G) 1EC diagnosed on an endometrial biopsy was referred to our Unit. She had a history of tuberculosis, HBV infection, and seropositive erosive rheumatoid arthritis associated with scleroderma. A total laparoscopic hysterectomy with bilateral salpingo-oophorectomy and pelvic bilateral systematic lymphadenectomy were performed: frozen section examination identified a 5 cm EC, deeply invading the myometrium. The final diagnosis was of an International Federation of Gynecology and Obstetrics stage IB, G 2 endometrioid EC (myoinvasion of 12 mm on 15 mm of myometrial thickness), showing lymphovascular invasion. Cervical stroma, parametria, Fallopian tubes, ovaries, 18 pelvic lymph nodes, and surgical margins were uninvolved. External radiotherapy (ERT) and vaginal brachytherapy (BRT) were administered.

**Figure 1:**
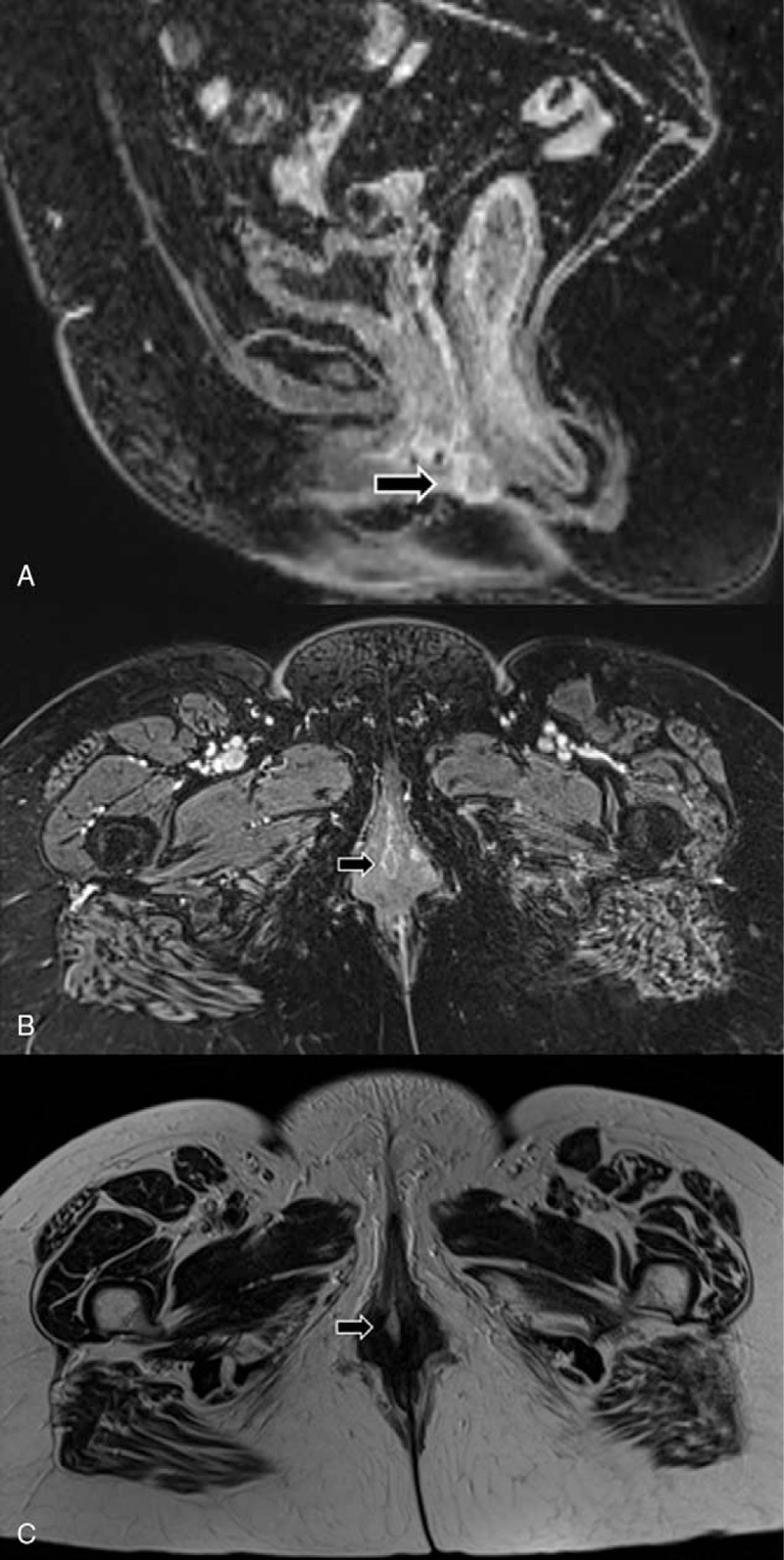
Magnetic resonance imaging (MRI). A, Sagittal contrast-enhanced T1-weighted MRI scan shows an enhancing mass (arrow) in vulva. B, Axial contrast-enhanced T1-weighted MRI scan shows enhancing mass (arrow) in right vulva. C, Axial T2-weighted MRI scan shows hyperintense mass (arrow) in right vulva.

**Figure 2:**
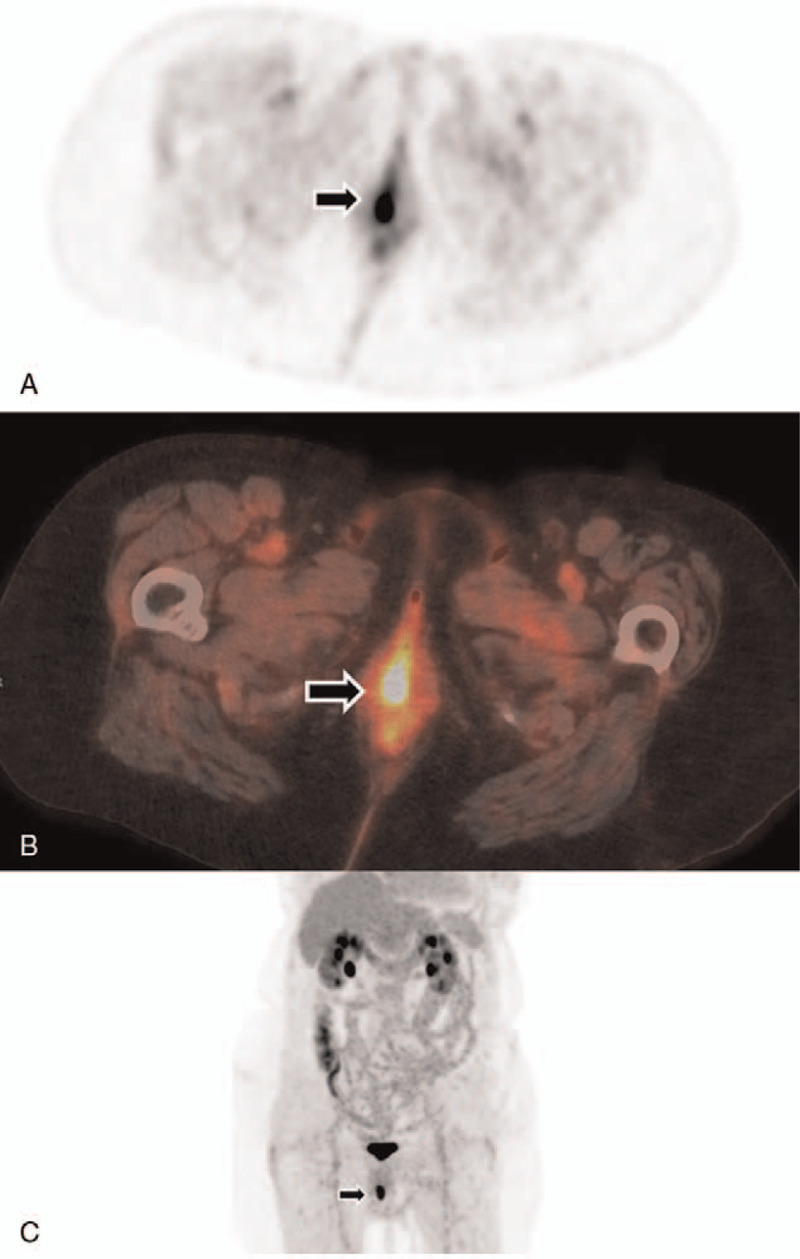
Positron emission tomography/computed tomography (PET/CT) identified a vulvar mass (arrow) with fluorodeoxyglucose (FDG)-avidity (arrow) (A, B, axial scans; C, coronal maximum-intensity-projection).

**Figure 3:**
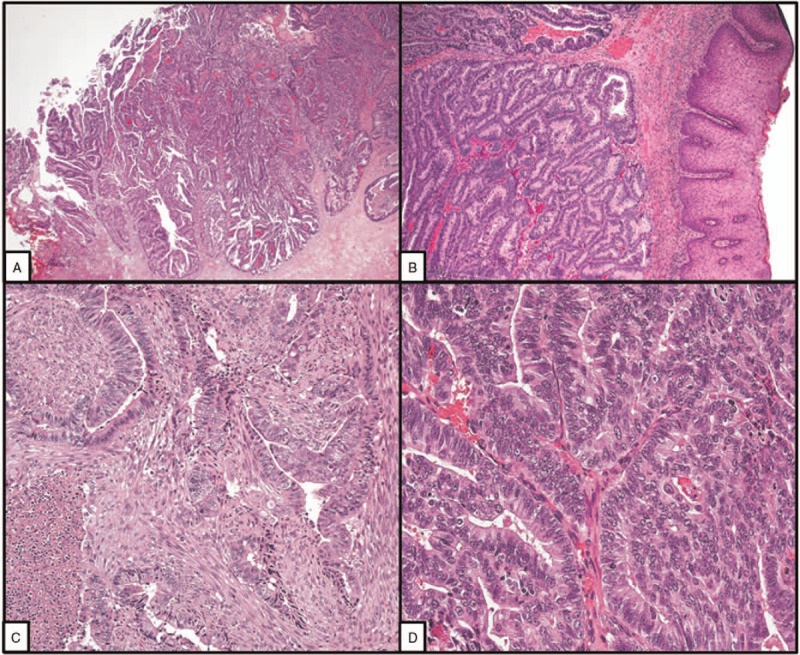
Histopathological features. A, Vulvar metastasis from endometrioid carcinoma of the endometrium (VM-EC). The metastatic carcinoma extensively replaced and ulcerated the vulvar mucosa; the tumor showed papillary, cribriform, and glandular histological patterns (hematoxylin and eosin, HE, ×4). B, VM-EC. In this area, the carcinoma (on the left) grew under the normal vulvar epithelium (on the right) (HE, ×10). C and D, Details. The myoinvasive primary endometrial carcinoma (C) and the vulvar metastasis (D) showed the same morphology. Glandular and cribriform patterns. Moderate nuclear atypia. (C, HE, ×20; D, HE, ×40).

**Figure 4:**
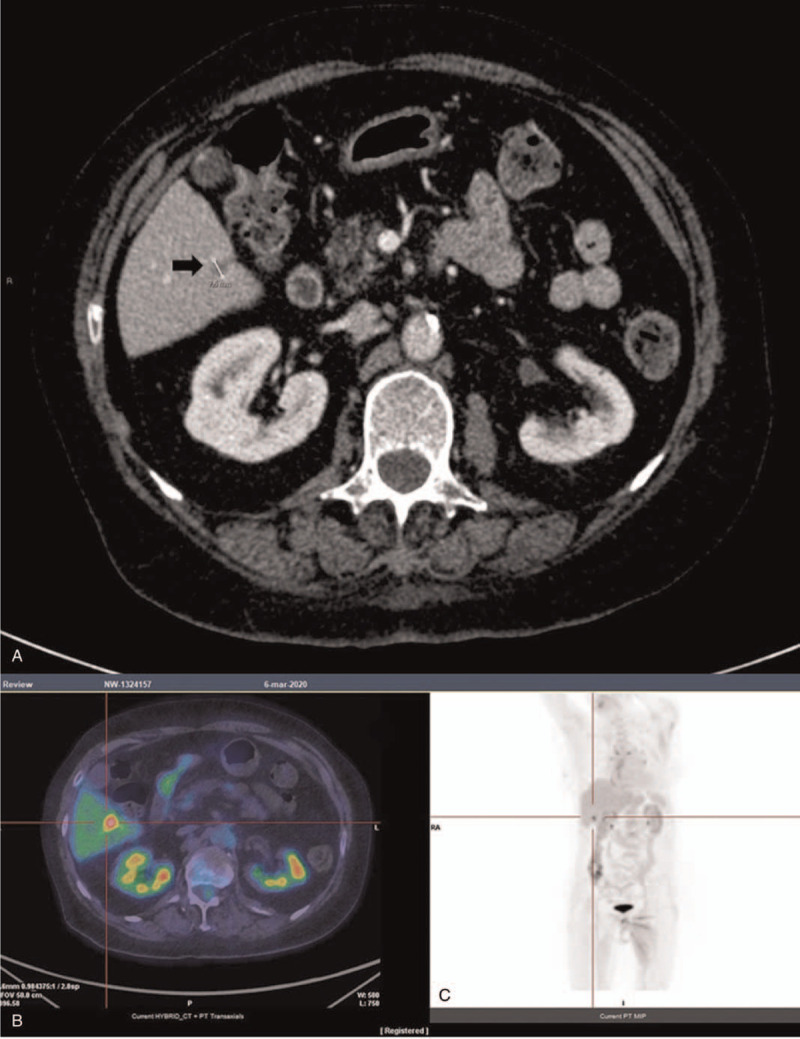
A, Axial contrast-enhanced T1-weighted MRI shows an enhancing hepatic mass (arrow); B and C, PET/CT images show an hepatic mass with FDG-avidity (arrows) (B, axial scan; C, coronal maximum-intensity-projection).

**Figure 5:**
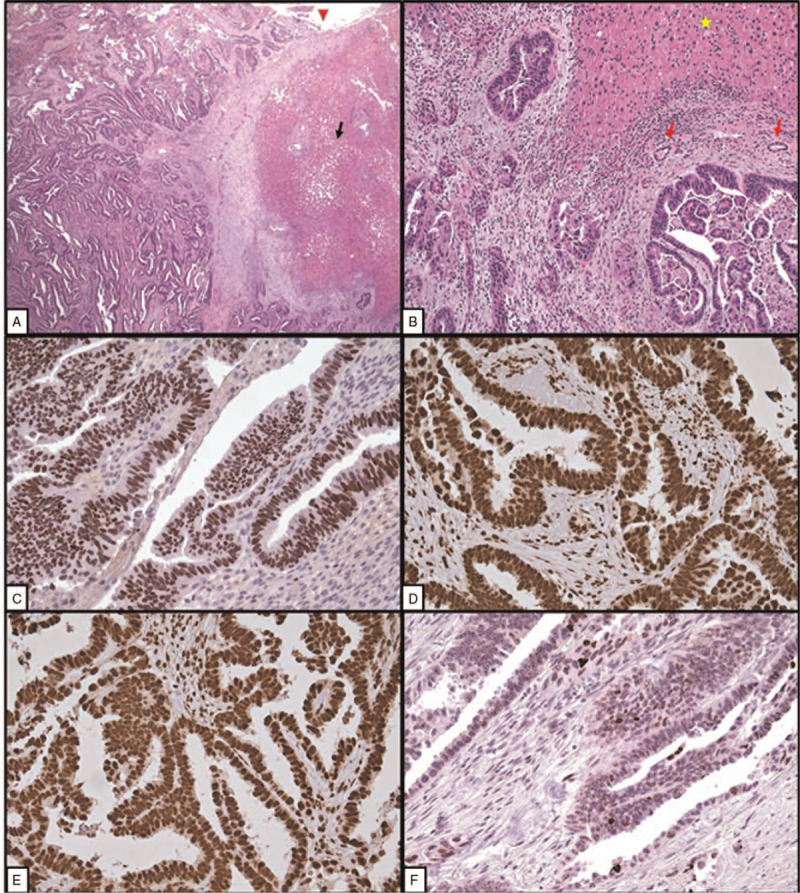
Hepatic metastasis from endometrioid carcinoma: histopathological features. A, The metastasis (on the left) involved the hepatic capsule (red arrowhead) and showed intraparenchymal growth. The hepatic parenchyma (on the right, black arrow) showed mild steatosis and reactive chronic inflammation (hematoxylin and eosin, HE, ×4). B, Detail of the tumor glands (bottom) (yellow star: hepatic parenchyma; red arrows: normal biliary ducts) (HE, ×20). C, Immunohistochemical nuclear positivity (strong, diffuse) of tumor cells for estrogen receptors (×10). D and E, Tumor cells showed retained (normal) immunohistochemical nuclear expression (strong, diffuse) of mismatch repair proteins (D, MLH-1, ×10; E, MSH-6, ×10). F, Wild-type expression of p53 in the tumor cells (rare cells are positive with variable intensity) (×10).

A 3 cm, reddish, bleeding lesion of the posterior commissura/right labia was found 11 months later: biopsy confirmed the EC recurrence. The lesion was weakly contrasted on pelvic magnetic resonance imaging (Fig. 1), with high standardized uptake value (5.2) on positron emission tomography (Fig. 2). The VM (G2 EC) was radically resected with free margins (Fig. 3). As imaging examinations excluded other metastases, the patient was followed up without additional treatment. Six months later, positron emission tomography (Fig. 4) showed only a 1.5 cm lesion with high uptake (standardized uptake value: 8.1) in the VI° hepatic segment: a wedge resection was laparoscopically performed. Histological examination confirmed an EC recurrence (G2 EC), focally invading the perihepatic fat; surgical margins were uninvolved (Fig. 5). On immunohistochemical examination (Supplementary material), tumor cells expressed estrogen receptor (75%), lacking expression of progesterone receptor. p53 expression resulted wild-type according to established criteria.^[15]^ The mismatch repair system proteins (MLH1; MSH2; MSH6; PMS2) resulted positive (normal expression). Six cycles of Carboplatin and Taxol were scheduled. After 2 cycles of chemotherapy, the patient died from a severe sepsis 27 months after primary surgery. IRB approval is not requested for “case report,” our patient signed standard consent for treatment of pseudonymized data, pictures, and videos for teaching and research purposes at the time of operation.

## 3 Review of literature

### 3.1 Systematic review of the literature

We performed a literature review of the EC VMs cases published from January 1966 to May 2020. Combinations of the terms “endometrial cancer” with “vulvar metastasis,” “vulvar recurrence,” “vulvar relapse,” or “vulvar spread” were searched in PubMed database, without setting limitations. Statistical analysis was performed using R-3.2.3 software. Quantitative variables are reported as mean ± standard deviation or median and interquartile range. The categorical variables were expressed in absolute frequency and percentage. OS was computed as the time period from the date of surgery to either the date of death or last follow-up.

### 3.2 Primary uterine ECs

Including our patient, 19 VM-cases were found^[5–14]^ (Table 1): a case lacking information was excluded.^[7]^ EC histotype was reported in 10/18 (55.5%) cases, including 1/10 (10%) serous,^[10]^ 1/10 (10%) clear cell,^[5]^ and 8/10 (80%) endometrioid ECs.^[5,8–14]^ Seven ECs were graded (G1: 3/7, 42.8%; G2: 2/7, 28.6%; G3: 2/7, 28.6%).^[8–14]^ International Federation of Gynecology and Obstetrics stage was reported in 8 of 18 (44.4%) cases: ECs frequently presented at stage IB (5/8, 62.5%),^[9,10,12,13]^ rarely at IA,^[11]^ IIIA,^[14]^ or IIIC^[8]^ (1/8, 12.5% each).

Treatment information was available for 11 of 18 (61.1%) cases.^[5,8–14]^ Three of 18 (16.7%) patients were treated by exclusive ERT+BRT (+ interstitial radiotherapy in 1 of 3 cases).^[5]^ Nine of 18 (50%) women underwent total abdominal hysterectomy ^[5,8–14]^ (+bilateral salpingo-oophorectomy in all except 1 case).^[10]^ Systematic pelvic and lombo-aortic lymphadenectomy was performed in 1 of 9 cases (11.1%),^[9]^ sampling of pelvic lymph nodes in 1 of 9 cases (11.1%),^[8]^ and pelvic/lombo-aortic lymph nodes sampling in 4 of 9 cases (44.4%).^[10,12–14]^ A patient received neoadjuvant ERT+BRT.^[5]^ In 12 of 18 (66.7%) patients, information about adjuvant therapy was reported^[5,8–14]^: 1 of 12 (8.3%) received ERT,^[11]^ 3 of 12 (25%) BRT,^[10,12,13]^ and 1 of 12 (8.3%) chemotherapy.^[14]^

### 3.3 VMs

Patients’ mean age was 66 years (range: 51–87 years). Labium major was the most common site of recurrence (9/17, 52.9%),^[5,6,10,11]^ followed by labium minus (2/17, 11.8%),^[6]^ introitus (2/17, 11.8%),^[6,9]^ posterior commissure (2/17, 11.8%),^[8,13]^ and clitoris (2/17, 11.8%).^[4,12]^ A VM extended to pubis/lower abdomen and vaginal vault.^[14]^ VMs were nodular/mass-like (9/18, 50%),^[5,6,8,9]^ exophytic (4/18, 22.2%),^[10,11,13]^ ulcerated (3/18, 16.7%),^[6,10]^ or papular (1/18, 5.5%),^[14]^ causing clitoromegally in 1 case (5.5%).^[12]^ Bleeding (3/18, 16.7%)^[5,9,10]^ and pain (4/18, 22.2%)^[5,14]^ were reported, but most of patients (61.1%) were asymptomatic.^[6,8,10–13]^

The mean size of VMs was 3.1 ± 2.6 cm (available information for 50% of cases).^[5,9–13]^ VMs were treated by radiotherapy (5/18, 27.8%),^[5,6]^ surgery (4/18, 22.2%),^[9–11,13]^ chemotherapy (2/18, 11.1%),^[6]^ surgery and radiotherapy (2/18, 11.1%),^[6,12]^ chemoradiation (2/18, 11.1%),^[6,14]^ surgery and chemoradiation (1/18, 5.5%),^[10]^ surgery and progestin therapy (1/18, 5.5%),^[8]^ or only follow-up (1/18, 5.5%).^[5]^

### 3.4 Follow-up

**Figure 6:**
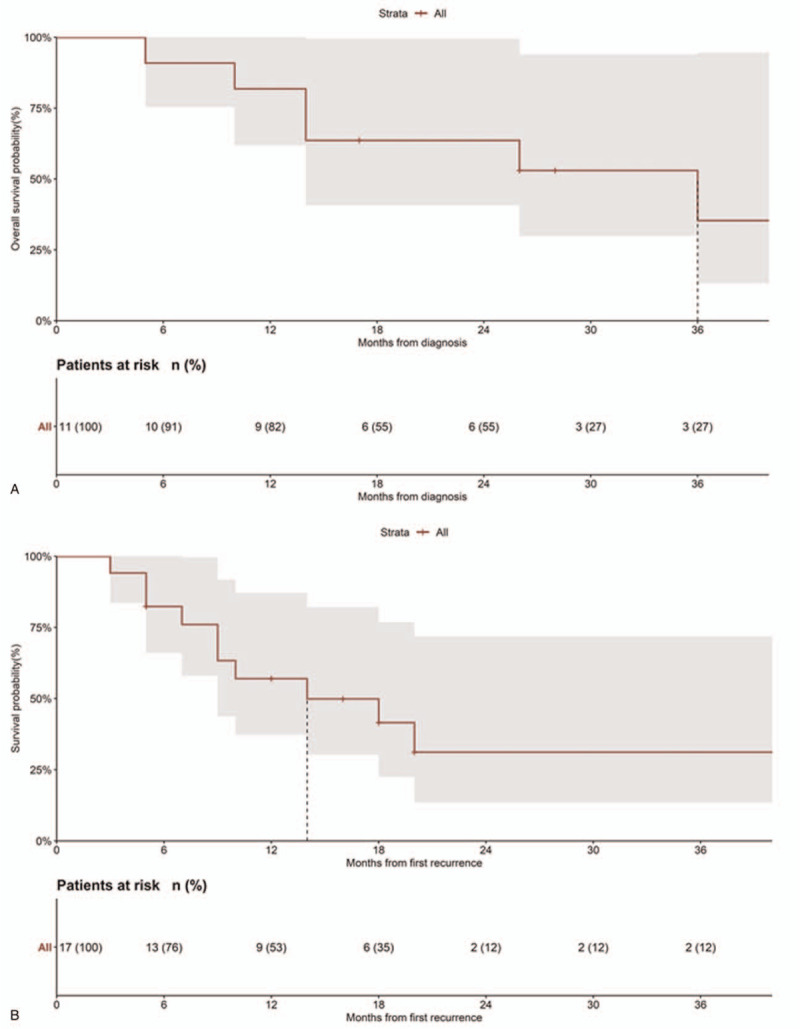
A, Overall survival of 18 patients with vulvar metastasis reported in the literature. B, Survival from vulvar metastasis treatment.

Follow-up information was reported in all the 18 patients, including time to VM in 11/17 (64.7%) cases (range 4–84 months; median 10 months).^[5,8–14]^ In 3 of 18 (16.7%) cases VMs were associated with recurrences in other sites.^[8,10,14]^ An EC with vaginal involvement presented with VM.^[5]^ An EC recurred on the vaginal cuff 5 months after primary treatment before relapsing on the vulva: the vaginal metastasis was treated with ERT+BRT.^[10]^ Six of 18 (33.3%) cases presented a subsequent recurrence after VM.^[5,10,12]^ Median time to subsequent recurrence was 10 months (range 5–14 months). Six of 18 (33.3%) patients were free of disease,^[6,9–11,13,14]^ 11 of 18 (61.1%) died of disease, ^[5,6,8,10,12]^ and 1 of 18 (5.6%) was alive with disease .^[6]^ Median OS was 16 months (5–104 months). The median OS from VM treatment was 14 months (3–84 months); 76.5% of patients were alive after 6 months, 53% were alive after 12 months, 35% after 18 months, 12% after 24 months (Fig. 6).

## 4 Discussion

Despite that EC is the most common gynecologic cancer in Western countries, VMs from ECs are very rare, only 19 cases were reported in literature in the last 50 years^[5–14]^ and only 1 case was reported in our hospital in the last 10 years.^[16,17]^ Like our case, VM is usually an isolated recurrence, being associated with other metastatic sites in 3 of 18 (16.7%) patients.^[8,10,14]^ VMs were mostly diagnosed in patients with low-intermediate risk ECs (66.7%)^[9–13]^: only 2 ECs presented at advanced stage (IIIA, IIIC).^[8,14]^ According to the stage, 7% to 15% of stage I to II ECs recur^[18,19]^: the relapse rate is higher for adjuvant irradiation (12.9% vs 7.2%) except for local recurrences (3.7%).^[18]^ As our case, 6 of 13 (46.1%) primary ECs received adjuvant radio^[5,11–13]^ or chemotherapy,^[14]^ and a VM appeared 5 months after irradiation of a vaginal cuff recurrence.^[10]^ VMs usually arose on labia majora as asymptomatic nodules/masses^[5,6,8–13]^; however painful bleeding exophitic/ulcerated/papular lesions were also reported.^[5,6,10,11,13,14]^ As for other sites frequently involved by EC recurrences, VMs were diagnosed within 2 years from primary treatment (median time: 10 months).^[3,19]^ VMs occurred most frequently in postmenopausal women, frequently representing a worrisome prognostic event: the median survival after VM was 20 months.^[20,21]^ The 3 year OS of distant recurring ECs varied from 14%^[15]^ to 54.3%.^[22]^ The 5-year OS was 55% for pelvic recurrences, being reduced to 17% for extrapelvic relapses.^[23]^ To our review, 61.1% (11/18) of VM patients died of disease ^[5–8,10,12,14]^: the maximum OS was 84 months.^[6]^ As other extrapelvic recurrences, VMs may be a prognostic risk factor^[3,16,23]^ causing rapid halving of survival 12 months after diagnosis. VMs may be due to direct tumor spread through vagina,^[10,14]^ which also represents the most frequently preoperatively contaminated site because of tumor bleeding.^[24,25]^ For Paget's “seed and soil” theory, a fertile environment (soil) favors the proliferation of implanted tumor cells (seed)^[25]^: tumor seeding and injuries of genital mucosa during surgery should be prevented. Some authors suggested using a bag during transvaginal specimen removal in patients with an inelastic, atrophic vagina: the posterior fourchette may be fragile and easily-traumatized from the passage of a large uterus.^[13]^ We do not use a bag in our routine also because there are not strong evidence to recommend it. EC recurrences may also occur on incisional abdominal wounds/scars of laparotomic or mini invasive primary surgery, usually due to microscopic tumor seeding^[26–28]^: EC seeding on Bartholin's gland incision during preoperative hysteroscopy can also justify VMs.^[9]^ Limited and conflicting data suggested that pneumoperitoneum may alter the peritoneal surfaces, favoring cancer cell adherence.^[24]^ However, it is certainly questionable to consider laparoscopy a risk factor for VMs as most patients were primary treated with laparotomy^[5,8–12,14]^ (1 with laparoscopy).^[13]^ Hematogenous or lymphatic spread may represent alternative pathways of tumor dissemination, like in our case: extensive radical pelvic surgery or radiotherapy can enhance lymphatic stasis with a possible retrograde spread of tumor emboli to vulvar lymphatics.^[5,8–11,12,14]^ There are lymphatic vessels that anastomose the pelvic and superficial inguinal lymphatic tributaries; moreover, uterine artery and both internal and external pudendal arteries are branches of the internal iliac artery with possible anastomoses or tumor emboli.^[24]^ Synchronous tumors may attract the implantation of EC cells, as suggested for a vulvar squamous cell carcinoma.^[11,25]^ Sometimes, it remains impossible to know the pathogenesis of VMs.

VMs can show different appearance (exophytic, nodular, papular, ulcerated), occurring as single or diffuse lesions on healthy or injured skin, in patients treated for both early- and advanced-stage ECs. Surgical approach seems not to influence the risk of subsequent VMs, but tumor seeding and vaginal injuries should be avoided. Whether isolated or associated with recurrence in other locations, VMs were characterized in most cases by a poor prognosis despite radical treatment. Therefore, any suspected vulvar lesion arisen during EC follow-up should be biopsied, despite that vulvar metastasis is unusual in EC patients.

## Author contributions

**Conceptualization:** Vincenzo Dario Mandato.

**Data curation:** Valentina Mastrofilippo.

**Formal analysis:** Valentina Mastrofilippo.

**Investigation:** Andrea Palicelli, Monica Silvotti, Silvia Serra, Lucia Giaccherini, Lorenzo Aguzzoli.

**Validation:** Monica Silvotti, Silvia Serra, Lucia Giaccherini.

**Writing – original draft:** Vincenzo Dario Mandato, Valentina Mastrofilippo, Andrea Palicelli, Lorenzo Aguzzoli.

## Supplementary Material

Supplemental Digital Content
