# Supplementary material for: Solitary vulvar metastasis from early-stage endometrial cancer: Case report and literature review
Source: Medicine (Baltimore). 2021 Jun 4;100(22):e25863. doi: 10.1097/MD.0000000000025863 (PMC8183741; doi:10.1097/MD.0000000000025863)
Supplement: Supplemental Digital Content [file medi-100-e25863-s001.doc]

**Supplementary material**

**Details of immunohistochemical antibodies**

Estrogen Receptor: clone SP-1, rabbit monoclonal, Ventana Medical Systems, Tucson, AR, US.

Progesterone receptor: clone 1E2, rabbit monoclonal, Ventana Medical Systems, Tucson, AR, US.

MLH1: clone M1, mouse monoclonal, Ventana Medical Systems, Tucson, AR, US.

MSH2: clone G219–1129, mouse monoclonal, Ventana Medical Systems, Tucson, AR, US.

MSH6: clone 44, mouse monoclonal, Cell Marque, Rocklin, CA, US.

PMS2: clone EPR3947, rabbit monoclonal, Ventana Medical Systems, Tucson, AR, US.

p53: clone DO-7, mouse monoclonal, Ventana Medical Systems, Tucson, AR, US.
